# Association Between ECG Findings on Presentation and Outcomes in Patients with Takotsubo Syndrome

**DOI:** 10.3390/jcm15010193

**Published:** 2025-12-26

**Authors:** Lihi Levi-Gofman, Shaul Atar, Dana Grosbard, Gassan Moady

**Affiliations:** 1 Azrieli Faculty of Medicine, Bar Ilan University, Safed 13100, Israel; lihilevi85@gmail.com (L.L.-G.); shaula@gmc.gov.il (S.A.); dana.grsbrd@gmail.com (D.G.); 2 Department of Cardiology, Galilee Medical Center, Nahariya 2222605, Israel

**Keywords:** takotsubo, ST elevation, cardiac function, outcome

## Abstract

**Background**: Takotsubo syndrome (TTS) may mimic acute coronary syndrome (ACS) by sharing a similar presentation, electrocardiogram (ECG) findings, and elevated troponin. Different ECG abnormalities may be encountered on presentation in patients with TTS, with ST elevation being the most common. In the current study, we aimed to evaluate the association of different ECG patterns on outcomes of patients with TTS. **Methods**: Patients hospitalized for TTS between 2018 and 2024 were included in the study. Demographic, echocardiographic, electrocardiographic, and laboratory parameters were obtained. Patients were classified according to ECG into two main groups, with and without ST segment elevation. We compared echocardiographic parameters and clinical outcomes between the two groups. **Results**: A total of 119 patients (mean age 70, 97% females) were included in the final analysis. The mean left ventricular ejection fraction (LVEF) was 45%, and 59 (50%) of the patients had ST segment elevation on ECG. Patients with ST elevation had lower LVEF, higher troponin levels, and a higher rate of complications related to heart failure (HF) and arrhythmia. After adjusting for potential confounders, only reduced LVEF was associated with an increased rate of complications. There was no difference in readmission rate between patients with or without ST elevation. **Conclusions**: Patients with TTS presenting with ST segment elevation exhibit more severe disease, reflected by lower LVEF and higher troponin levels, albeit without increased risk for readmission.

## 1. Introduction

Takotsubo syndrome (TTS) is a form of transient cardiomyopathy that typically occurs in older women following mental or physical stressors [1]. Echocardiography plays a central role in the diagnosis, since the syndrome is typically characterized by unique wall motion abnormality with apical akinesia and basal hypercontractility known as apical ballooning [2]. TTS may mimic acute myocardial infarction (AMI), as it presents with chest pain, ECG changes, elevated troponin level, and cardiac wall motion abnormality [3,4,5]. Cardiac function recovery is typically rapid and complete, but the timeline varies. Many patients show substantial improvement within 48–72 h, but full normalization of LVEF often takes 7–14 days in uncomplicated cases and up to 4–8 weeks in others [6,7,8,9]. Traditionally, the MAYO criteria are commonly used to establish the diagnosis of TTS based on transient cardiac dysfunction, absence of coronary plaque rupture, new ECG abnormality or biomarker elevation, and exclusion of pheochromocytoma and myocarditis [4]. The newer interTAK diagnostic criteria highlight that TTS can coexist with coronary artery disease, the preceding trigger may be physical, emotional, or absent, cardiac function always recovers, ECG changes may include QT prolongation, and right ventricular involvement may occur [5]. Clinical course and treatment are mainly driven by the extent of left ventricular ejection fraction (LVEF) impairment [1,10,11]. Although most cases have a benign course with no significant complications, fulminant cases with malignant arrhythmia, acute heart failure (HF), and cardiogenic shock may occur [1,12,13,14]. Several biomarkers play a central role in the pathophysiology of TTS, and alteration in their levels may be used for diagnostic and prognostic purpose [15,16,17,18]. ECG changes in TTS may mimic AMI, since ST segment elevation in the precordial leads without reciprocal ST depression is common [19,20,21,22,23]. The evolution of ST segment changes in TTS is different from that in AMI, and there are several rules in use to differentiate between the two conditions [24,25,26]. In TTS, ST elevation appears immediately or within hours from symptoms onset, and after 1–3 days, T wave inversion may be present after ST segment resolution [24,25,27]. After several weeks, the T wave may be deeper or normalize, generally without Q wave development [24,25,26,27]. Similar to acute ischemia, QT segment prolongation in TTS is also not uncommon at presentation [28,29,30]. In the current study, we aimed to assess the prevalence of ST segment elevation in TTS and to test the hypothesis of whether it is associated with poor outcomes compared to patients with TTS and other ECG changes.

## 2. Materials and Methods

### 2.1. Study Population

This retrospective study is based on data of patients hospitalized with TTS. A total of 119 patients were identified based on the ICD-9 diagnosis of “Takotsubo syndrome” in the cardiology unit in the period 2018–2024. Diagnosis was confirmed based on clinical presentation, laboratory, echocardiography, and cardiac catheterization findings. Patients with suspected TTS based on clinical presentation, elevated troponin, and wall motion abnormality in echocardiography underwent coronary angiography to exclude left anterior descending artery stenosis/obstruction. In a few cases, when a patient refused angiography, TTS was highly suggested by typical echocardiographic appearance and clinical scenario. Once TTS was confirmed, a thorough second interview was usually performed to identify potential mental or physical stressors before clinical onset. Patients with signs of pulmonary congestion were treated with diuretics. Beta blockers and renin–angiotensin–aldosterone system inhibitors were used when cardiac dysfunction was observed, particularly when accompanied by HF signs. In cases of cardiogenic shock secondary to left ventricular outflow tract (LVOT) obstruction, discontinuation of inotropes and administration of intravenous beta blockers and fluids were applied. All patients had an apical ballooning pattern (typical TTS). We did not encounter other patterns, such as inverted TTS or focal TTS, probably due to the rare incidence of these patterns or to underdiagnosis.

The study was approved by the local ethics committee. The approval number was NHR-0073-24; the approval date was 17 April 2024.

### 2.2. Laboratory Parameters

For each patient, complete blood count, renal function, and troponin level were obtained. High-sensitivity troponin I (hs-TnI) levels were measured using ARCHITECT assay (Abbott Laboratories, Abbot Park, IL, USA). Cut-off values for abnormal hs-TnI levels were above 20 ng/L and 30 ng/L for men and women, respectively.

### 2.3. Echocardiography

Echocardiography was performed using a Philips Epiq-7 machine with EPIQ X8-2t transducer (Phillips, Adnover, MA, USA). LVEF was calculated by Simpson’s biplane method in the apical four-chamber and two-chamber views using the following formula: LVEF = Left ventricular end diastolic volume-Left ventricular end systolic volume)/Left ventricular end diastolic volume X100. A typical echocardiographic appearance includes apical hypokinesia/akinesia and basal hypercontractility. All echocardiographic studies were performed and interpreted by an expert cardiologist.

### 2.4. Electrocardiographic Data

Electrocardiograms (ECGs) were analyzed upon admission. Based on ECG findings, patients were categorized into two groups: those with anterior ST-segment elevation (leads V1–V4) and those without ST-segment elevation. The latter group included individuals exhibiting ST-segment depression, T-wave inversion, complete bundle branch block, or non-specific ECG changes.

### 2.5. Outcomes

Complications during the index hospitalization included arrhythmia (both atrial and ventricular) and pulmonary congestion. LVOT obstruction was reported separately. Mortality during the index hospitalization and readmission rates within one year were also reported. We did not perform serial echocardiography, except for a few cases of severe LV dysfunction or cardiogenic shock. Follow-up echocardiography was documented in 89 patients (75%).

### 2.6. Statistical Analysis

Categorical variables are presented as percentages, whereas continuous variables are presented as means with standard deviations (SDs) or medians with interquartile range (IQRs). The Mann–Whitney test and an independent *t* test were applied to evaluate the correlation between continuous variables, while the chi square test was used for categorical variables. Multivariable logistic regression was performed to adjust for possible confounders. Kaplan–Meier curves were used for assessing readmissions. All statistical analyses were performed using IBM SPSS version 27.

## 3. Results

Our cohort included 119 patients (94% female, mean age 70 years). The main presenting symptom was chest pain, and it was preceded by a physical or mental trigger in 74% of the cases. The baseline characteristics and background diseases are summarized in Table 1.

During the index hospitalization, patients were treated in accordance with their clinical presentation and cardiac function. Upon admission, 59 (50%) patients had ECG changes, and the mean LVEF was 45%. ECG was performed within a median time of 4.5 h after symptom onset. Complications, including arrhythmia and HF symptoms, were reported in 18% of patients. It should be noted that most arrhythmias were not malignant and resolved spontaneously without hemodynamic compromise. One patient had symptomatic ventricular tachycardia treated with electrical shock. Supraventricular arrhythmia in TTS typically resolves spontaneously. Dynamic LVOT obstruction (with mitral regurgitation (MR) secondary to systolic anterior motion of the mitral valve) was reported in 12 patients (10%). The severity of MR was mild in most cases. In only three cases, the degree of MR was severe. Patients with LVOT obstruction were treated with beta blockers and intravenous fluids (and discontinuation of inotropes). One patient passed way due to cardiogenic shock. Table 2 summarizes the main hemodynamic and laboratory parameters.

**Figure 1:**
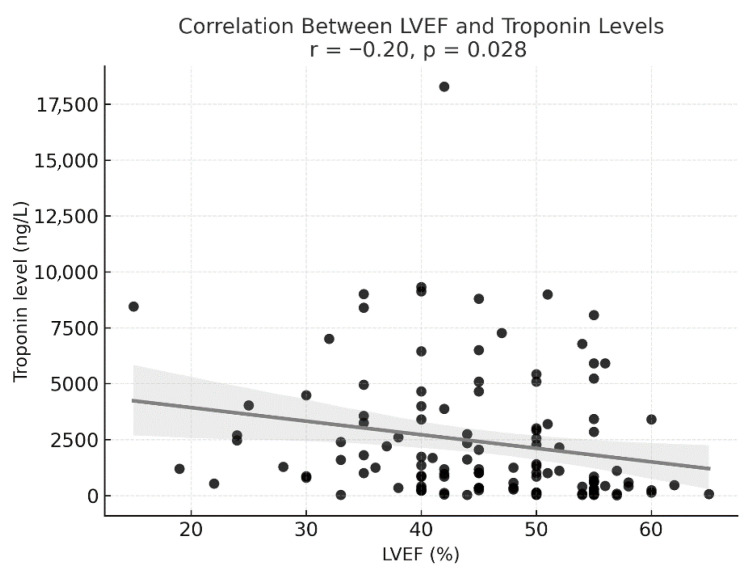
Correlation between LVEF and troponin levels.

Patients with lower LVEF had higher levels of troponin during the index hospitalization. The correlation is depicted in Figure 1.

Figure 1 shows an inverse correlation between LVEF and troponin levels. Although TTS is generally not associated with extremely high troponin levels, a weak correlation was demonstrated in our study, reflecting the extent of myocardial stress.

Patients with ST segment elevation had lower LVEF and higher troponin levels (*p* = 0.003 and *p* = 0.014, respectively). The complication rate related to HF and arrhythmia was higher in the ST elevation group (*p* = 0.01), whereas the rate of dynamic LVOT obstruction was similar (*p* = 0.21). Comparison between the two groups is given in Table 3.

**Figure 2:**
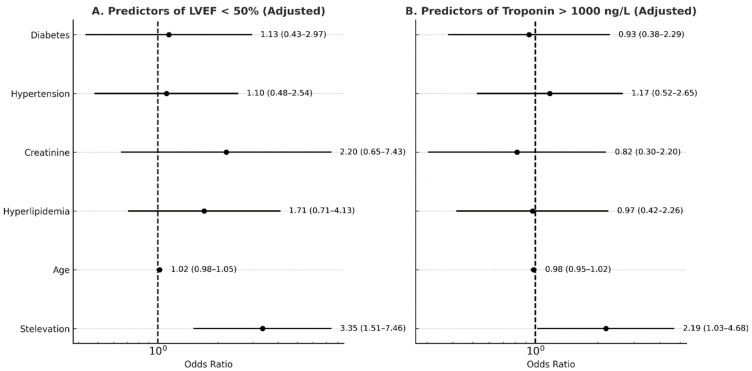
Summary of multiple logistic regression for predicting reduced LVEF (**A**) and high troponin levels (**B**).

In the multivariable logistic regression adjusted for age, hyperlipidemia, hypertension, diabetes, creatinine, and hemoglobin, ST elevation was independently associated with a significantly increased likelihood of cardiac dysfunction (defined as LVEF < 50%) and high troponin levels, as shown in Figure 2A and Figure 2B, respectively.

When adjusting for age, hypertension, hyperlipidemia, diabetes, and creatinine levels, ST elevation remained independently associated with an increased risk of reduced LVEF (A) and higher troponin levels (B), defined as levels above 1000 ng/L.

**Figure 3:**
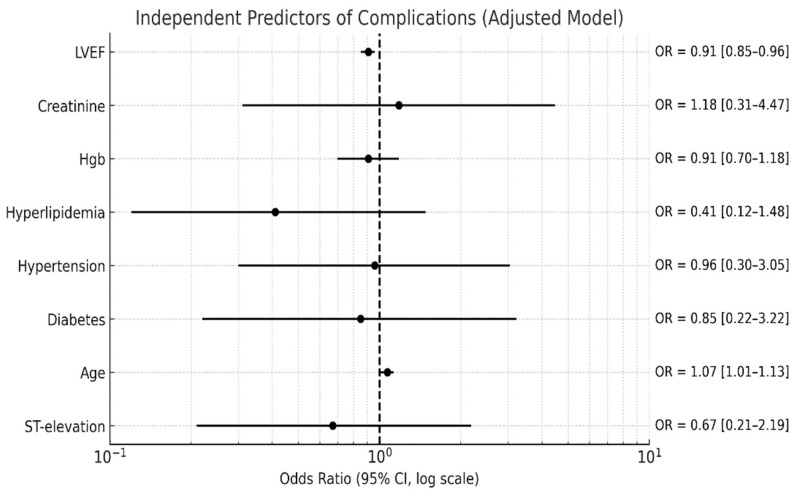
Multiple logistic regression for predicting in-hospital complications. Hgb, hemoglobin; LVEF, left ventricular ejection fraction, OR, odds ratio.

In-hospital complications were more common in the ST elevation group (27% vs. 8%, *p* = 0.01). After adjustment for potential confounders (LVEF, age, hyperlipidemia, diabetes, hypertension, creatinine, and hemoglobin levels), reduced LVEF and older age were the only independent predictors (Figure 3).

After adjusting for other confounders, only reduced LVEF and older age remained independently associated with an increased risk of in-hospital complications.

**Figure 4:**
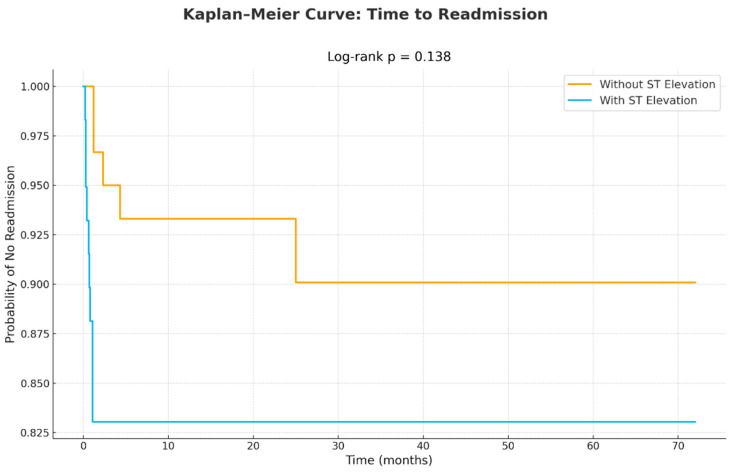
Kaplan–Meier curve for readmissions in the two groups.

The readmission rate was similar between patients with and without ST elevation, as illustrated in Figure 4. Readmissions were mainly for chest pain (about 80%), and about 20% of readmissions were for HF-related symptoms such as pulmonary congestion. Recurrent TTS was reported in two cases (both in the ST elevation group). All recurrent hospitalizations had a benign course. Follow-up echocardiography was available for 89 patients (75%), all of whom demonstrated complete recovery of cardiac function. Electrocardiographic follow-up was unavailable for most patients; however, it is well established that ECG abnormalities in TTS typically resolve and return to baseline during recovery.

The KM curve in the figure shows admissions for heart failure symptoms. There was no difference between the two groups (*p* = 0.138).

## 4. Discussion

In the current study, we evaluated the association between ST segment elevation upon admission and outcomes of patients with TTS. We found that patients with ST segment elevation in the precordial leads have higher troponin levels and lower LVEF. After adjusting for potential confounders, older age and poor LVEF were associated with more in-hospital complications, including HF and arrhythmia. The rate of dynamic LVOT obstruction was similar between the two groups. There was no difference in readmission rates between patients with or without ST elevation. ECG abnormalities are very common in patients with TTS; among them, ST segment elevation in the precordial leads is most frequently encountered in the acute phase [31]. Theoretically, ST elevation may indicate more extensive wall motion abnormality in severe cases. However, studies that investigated the correlation between ST segment elevation and outcomes showed conflicting results [31,32]. Unlike ST elevation, marked QT prolongation is well established as a marker for increased risk of torsade de points and sudden cardiac death in TTS [28,29,30]. Repolarization abnormalities such as deep T wave inversion in the precordial leads have also been suggested as an independent risk factor, but the data is also conflicting [33,34,35]. In the International Takotsubo Registry, a variety of prognostic factors were identified for TTS, such as reduced LVEF, cardiogenic shock, a physical stress trigger (versus an emotional one), male sex, and neurological comorbidity [1]. We found that ST elevation was associated with higher troponin levels and poor LVEF. Similar to AMI, ST elevation and repolarization abnormalities in TTS may actually serve as a risk marker since they correlate with the degree of cardiac dysfunction. The presence of repolarization changes in TTS signals myocyte dysfunction and myocardial edema, even in the absence of major necrosis [36]. Since TTS is characterized by transient wall motion abnormalities and myocardial stunning rather than infarction, the repolarization changes may reflect stunning and microvascular dysfunction rather than irreversible damage. The correlation between troponin levels and LVEF in TTS also differs from AMI. In TTS, troponin levels are generally modestly elevated and may reflect stunning in the acute phase or direct myocyte injury following the catecholamine surge accompanying TTS, while natriuretic peptide levels are often extremely elevated, making the high ratio of NTproBNP/troponin very unique to TTS, which may help to distinguish it from AMI [15,16,17,37,38]. As already established, troponin levels show only a weak inverse correlation with LVEF among patients with TTS. The degree of cardiac dysfunction in TTS is primarily driven by catecholamine-induced myocardial stunning and microvascular spasm rather than direct myocyte necrosis. Troponin elevation in TTS is usually modest. It should be noted that patients with severely reduced LVEF may still have only modest troponin release, reflecting reversible injury rather than the myocardial necrosis seen in AMI. Complications related to HF symptoms and arrhythmia were more common in the ST elevation group in our cohort. Acute HF is the most common complication in TTS, followed by ventricular arrhythmia [1]. Other, less frequent complications are cardiogenic shock, left ventricular thrombus and systemic emboli, and mechanical complications (wall rupture or acute mitral regurgitation) [1]. However, after adjustment for other confounders, only age and LVEF were associated with an increased risk of complications. In a large analysis of TTS, patients ≥ 65 yrs had higher in-hospital mortality, more frequent acute kidney injury, and longer hospital stays than younger ones [39]. Older age was also related to long-term all-cause mortality and late cardiac recovery [40,41,42]. It should be noted that other studies showed inconsistent results. For example, in a study by El-Battrawy et al., lower mortality rates were observed in younger patients but with increased in-hospital complications due to atypical presentation [43]. These differences may be explained by different study populations and trends in diagnostic approaches and therapies over the years. One of the most potentially fatal complications of TTS is dynamic LVOT obstruction. We did not find a difference in the rate of LVOT obstruction between the two groups. Dynamic LVOT obstruction is mainly due to basal hypercontractility and is not necessarily related directly to the degree of cardiac dysfunction. Readmission rates were similar between the two groups in our cohort. Cardiac dysfunction usually resolves within days after the acute episode. Patients with TTS and signs of HF are usually treated with neurohormonal medications, mainly beta blockers and angiotensin-converting enzyme inhibitors. Patients may be safely discharged once there is no sign of hemodynamic compromise or volume overload or arrhythmia. Therefore, readmissions in TTS for HF signs may occur only during the first weeks following discharge. In summary, ECG alone should not be used in isolation for prognostication in TTS. A more abnormal ECG should prompt closer monitoring and possibly more aggressive supportive measures. ST elevation should be integrated with imaging, biomarkers, and clinical presentation for risk stratification in TTS. Implementation of natriuretic peptides is probably advised in all patents with TTS.

## 5. Limitations

Our study has several limitations. First, the study population was relatively small, and the majority of our patients were stable, without major complications, making our conclusions limited to this population. Second, the study was retrospective. Although we performed a multivariate regression, other confounders may still have affect the results of the study. Third, the evolution of ECG in TTS may vary between patients; however, we performed an ECG upon presentation to minimize such variations. Fourth, we did not have data about natriuretic peptides in all patients and, therefore, we did not include it in the analysis. Natriuretic peptides can serve as a strong predictor of outcomes, and their implementation in future studies may reinforce the results. Fifth, we did not divide the patients according to the type of TTS or by the type of trigger (physical vs. emotional). Sixth, our study included only patients with a typical apical ballooning pattern, making the results not applicable for other types, such as inverted-, midventricular-, or focal-type TTS.

## 6. Future Implications

Risk stratification in patients with TTS should always be dictated by the clinical course and echocardiographic findings. Patients with ST elevation (even without QT prolongation) may benefit from early transfer to the intensive care unit and close follow-up of cardiac function. Future scores for risk assessment should incorporate biomarkers and ECG changes, along with imaging findings and clinical presentation.

## 7. Conclusions

The presence of ST segment elevation upon presentation in patients with TTS may predict poor LVEF, with higher troponin levels indicating severe disease. However, ST elevation was not associated with readmissions.

## Figures and Tables

**Table 1:** Baseline characteristics of the patients.

*n*	119
Age, years (mean ± SD)	70 ± 12
Female (*n*, %)	112 (94)
Hypertension (*n*, %)	71 (60)
Diabetes mellitus (*n*, %)	37 (31)
Hyperlipidemia (*n*, %)	61 (51)
Tobacco use (*n*, %)	21 (18)
Chronic kidney disease (*n*, %)	40 (34)
Ischemic heart disease (*n*, %)	22 (18)
Neurological disease (*n*, %)	10 (8)
Psychiatric disease (*n*, %)	15 (13)
Presenting symptom	
Chest pain (*n*, %)	76 (64)
Dyspnea (*n*, %)	30 (25)
Other (*n*, %)	13 (11)
Trigger (*n*, %)	88 (74)

SD, standard deviation. Chronic kidney disease was defined as estimate glomerular filtration rate < 60 mL/min/1.73 m^2^.

**Table 2:** Hemodynamic and laboratory parameters of the study population.

*n*	119
LVEF % (mean ± SD)	45 ± 10
ECG changes (*n*, %)	59 (50)
Time, hours: symptom onset to ECG (median, IQR)	4.5 (2–5.5)
Troponin, ng/L (median, IQR)	1186 (340–3400)
CRP, mg/L (median, IQR)	15 (5–33)
Systolic BP, mmHg (mean ± SD)	123 ± 23
Diastolic BP, mmHg (mean ± SD)	73 ± 14
Heart rate, BPM (mean ± SD)	83 ± 16
WBCs, ×10^3^/μL(mean ± SD)	10 ± 4
Hemoglobin, gr/dL (mean ± SD)	12 ± 2
Creatinine, mg/dL (mean ± SD)	0.97 ± 0.4
Complications, HF and arrhythmia (*n*, %)	21 (18)
Dynamic LVOT obstruction (*n*, %)	12 (10)
1-year total readmissions	15 (13)
Mortality (*n*, %)	1 (1)

BP, blood pressure; BPM, beats per minute; CRP, C-reactive protein; ECG, electrocardiogram; IQR, interquartile range; LVEF, left ventricular ejection fraction; LVOT, left ventricular outflow tract; WBCs, white blood cells.

**Table 3:** Comparison between patients with and without ST elevation.

*n*	With ST Elevation 59	Without ST Elevation 60	*p* Value
Age, years (mean ± SD)	71 ± 12	69 ± 12	0.37
Hypertension (*n*, %)	36 (61)	36 (60)	1.0
Diabetes mellitus (*n*, %)	20 (34)	17 (28)	0.65
LVEF % (mean ± SD)	42 ± 10	48 ± 8	0.003 *
WBCs (×10^3^/μL)	10.3 ± 4.7	10 ± 4.6	0.77
Hemoglobin, gr/dL (mean ± SD)	11.5 ± 2.3	11.9 ± 2.2	0.34
Creatinine, mg/dL (mean ± SD)	1 ± 0.4	0.9 ± 0.4	0.33
Troponin, ng/L (median, IQR)	2045 (574–4263)	911 (226–2403)	0.014 *
CRP, mg/L (mean ± SD)	14 (5–32)	15 (7–27)	0.44
Complications, HF and arrhythmia (*n*, %)	16 (27)	5 (8)	0.01 *
Dynamic LVOT (*n*, %)	8 (14)	4 (7)	0.21
Total readmissions	10 (17)	5 (8)	0.138

* statistically significant *p* values.

## Data Availability

The data presented in the study are available upon request from the corresponding author, G.M.
